# Graphene functionalized field-effect transistors for ultrasensitive detection of Japanese encephalitis and Avian influenza virus

**DOI:** 10.1038/s41598-020-71591-w

**Published:** 2020-09-03

**Authors:** Akanksha Roberts, Neha Chauhan, Saurav Islam, Subhasis Mahari, Bhaskar Ghawri, Ravi Kumar Gandham, S. S. Majumdar, Arindam Ghosh, Sonu Gandhi

**Affiliations:** 1 DBT-National Institute of Animal Biotechnology (NIAB), Hyderabad, Telangana 500032 India; 2 Department of Physics, Indian Institute of Science (IISc), Bangalore, 560012 India; 3 Center for Nanoscience and Engineering, Indian Institute of Science (IISc), Bangalore, 560012 India

**Keywords:** Graphene, Nanobiotechnology

## Abstract

Graphene, a two-dimensional nanomaterial, has gained immense interest in biosensing applications due to its large surface-to-volume ratio, and excellent electrical properties. Herein, a compact and user-friendly graphene field effect transistor (GraFET) based ultrasensitive biosensor has been developed for detecting Japanese Encephalitis Virus (JEV) and Avian Influenza Virus (AIV). The novel sensing platform comprised of carboxy functionalized graphene on Si/SiO_2_ substrate for covalent immobilization of monoclonal antibodies of JEV and AIV. The bioconjugation and fabrication process of GraFET was characterized by various biophysical techniques such as Ultraviolet–Visible (UV–Vis), Raman, Fourier-Transform Infrared (FT-IR) spectroscopy, optical microscopy, Scanning Electron Microscopy (SEM) and Atomic Force Microscopy (AFM). The change in the resistance due to antigen–antibody interaction was monitored in real time to evaluate the electrical response of the sensors. The sensors were tested in the range of 1 fM to 1 μM for both JEV and AIV antigens, and showed a limit of detection (LOD) upto 1 fM and 10 fM for JEV and AIV respectively under optimised conditions. Along with ease of fabrication, the GraFET devices were highly sensitive, specific, reproducible, and capable of detecting ultralow levels of JEV and AIV antigen. Moreover, these devices can be easily integrated into miniaturized FET-based real-time sensors for the rapid, cost-effective, and early Point of Care (PoC) diagnosis of JEV and AIV.

## Introduction

The development of Point of Care (PoC) disease detection kits providing ultra-sensitive, selective, and rapid advances in recent times. In this article, we have focused on graphene-based biosensors for the detection of two different viruses by detecting their respective viral antigen i.e. Japanese encephalitis virus (JEV) and Avian Influenza Virus (AIV). JEV belongs to the family *Flaviviridae* genus Flavivirus^1^ and exists in a zoonotic cycle between the vector i.e. *Culex* mosquitos, while humans are the dead end host due to low and short-lived viremia of JEV^2–5^. Most infections of JEV are asymptomatic, however, the case-fatality rate among those with encephalitis can be as high as 30%, or more in children. It causes clinical symptoms in humans, including a non-specific febrile illness, meningitis, encephalitis and meningo-encephalitis. Pigs play an important role and serve as an amplifier and have a natural infection rate of 98–100%^6^. As JEV is incurable and the vaccination is not full-proof, an early diagnosis is critical in preventing an epidemic outbreak, especially since the initial symptoms are usually mistaken for dengue or malaria. The conventional diagnostic methods for JEV^7^ such as Enzyme-Linked Immunosorbent Assays (ELISA)^8^, Reverse Transcriptase Polymerase Chain Reaction (RT-PCR)^9^, Reverse Transcription Loop-mediated Isothermal Amplification (RT-LAMP) assay^10^, Luminex Technology^11^, Plaque Reduction Neutralisation Test (PRNT)^12^, Hemagglutination Inhibition (HI) test^13^, Complement Fixation Test (CFT)^14^, Immunofluorescence Test (IFT)^15^, and virus isolation^16,17^ are costly and time-consuming diagnostic/assay procedures that require bulky equipment and trained personnel.

AIV is a disease caused by infection of avian influenza type A virus from *orthomyxoviridae* family, transmitted through direct contact, contaminated surfaces or from the viral droplets in air. This disease causes major loss to the poultry industry which is the largest source of meat and eggs for humans. Diagnosis of AIV begins upon the visibility of the symptoms starting with isolation of live virus from embryonated chicken eggs but this method is time consuming and laborious^18^. Other methods of diagnosis include virus subtyping by H1 and neuraminidase inhibition tests^19^, subtyping by RT-PCR^18^, real time Polymerase Chain Reaction (PCR)^20^, nucleic acid based amplification^21^, next generation sequencing^22^, and immunochromatography^23^. The diagnostic tools discussed above are costly and require trained professionals.

A portable diagnostic technique is required that can provide rapid results at the onset of the disease when the antigen concentration is very low^24^. In recent times, immunobiosensors/biochips have begun to replace the conventional methods^25–30^. Electrochemical biosensors have received much attention as a reliable diagnostic tool for infectious diseases as their sensing performances are not affected by turbidity or absorbance of the sample^31^. Moreover, electrochemical biosensors offer the advantages of being highly sensitive, rapid signal generation, easy to miniaturize, and inexpensive^32^. Field effect transistors (FET), sensitive to the local charge environment, can be utilized for real-time study of the target-analyte interaction that causes a change in the channel resistance/conductance and can be easily monitored. Such FETs made of graphene have been investigated extensively in recent years.

Graphene is made up of a single layer of sp^2^ bonded carbon atoms arranged in a two-dimensional honeycomb network, displaying very high electron mobility, excellent electrical conductivity, tunable optical properties, and high mechanical strength to name a few^33^. A high surface to volume ratio, also ensures that it can absorb a large quantity of aromatic biomolecules by π–π stacking^33^, making it an ideal nanomaterial^34,35^ which can be used as a platform for a wide range of biological applications^36^. It has been used for the detection of various types of biomolecules such as Deoxyribonucleic Acid (DNA), enzymes, antibodies, aptamers, etc.^37–40^. Another advantage of using graphene is its efficient covalent conjugation of Ab with graphene using different chemicals such as polyethylene glycol (PEG)^41,42^, EDC-NHS^43–47^ etc. Based on previous literature^29,43–47^, it is known that EDC-NHS carbodiimide conjugation created strong covalent bonds which were not affected on addition of other components in further experimental steps and hence in this paper we selected EDC-NHS carbodiimide reaction for activation and conjugation of graphene.

In this study, to the best of our knowledge for the first time, we have demonstrated a graphene-based field-effect transistor (GraFET) for the detection of JEV and AIV antigen. The amine functionalized antibodies anti-JEV for JEV and anti-AIV for AIV were covalently coupled with carboxyl activated graphene. The binding was ensured at each step of functionalization, antibody (Ab) attachment, and capture of antigen (Ag) by various techniques such as UV–Vis, FT-IR, Raman spectroscopy, optical imaging, and SEM. The limit of detection (LOD) for JEV and AIV was confirmed by adding different concentration of antigens from 1 fM to 1 µM separately, and monitoring the change in resistance of the graphene channel as a function of time. The obtained results demonstrate that the developed GraFET device can be successfully employed in Point of Care diagnostics on field after miniaturization process and can also be applicable for the detection of other infectious diseases as well.

## Results and discussion

### Characterization of graphene-bioconjugate

**Figure 1:**
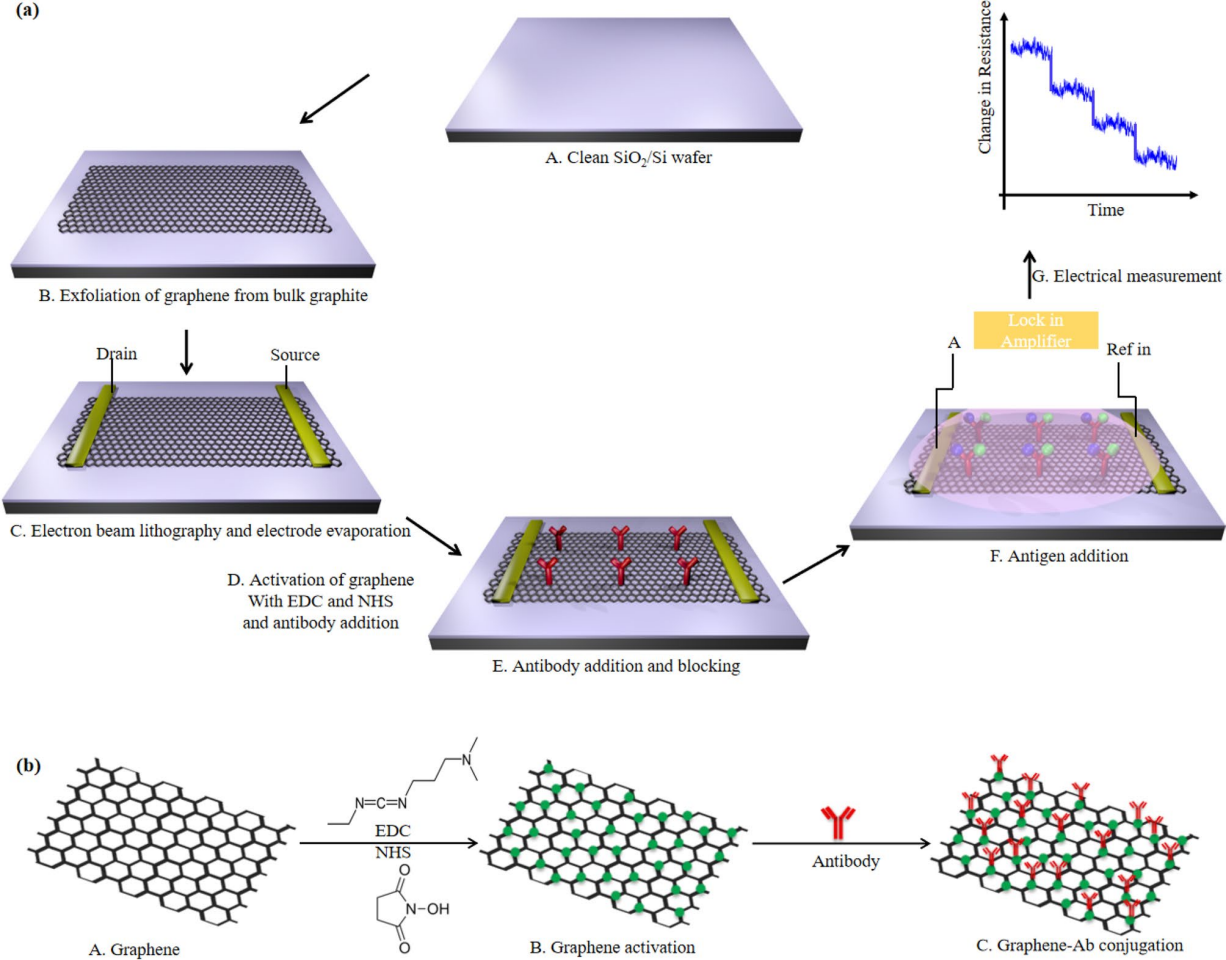
**(a)** Schematic representation of the steps involved in the fabrication of GraFET biosensor: **(A, B)** Graphene was exfoliated on pre-cleaned Si/SiO_2_ substrate using 3 M scotch magic tape. **(C, D)** E-beam lithography was used to define the source and drain pads followed by thermal evaporation of 5/50 nm Cr/Au by which graphene was electrically contacted **(E, F)** Graphene was activated using EDC-NHS carbomiide reaction and functionalised by drop casting Ab on the channel. BSA used as the blocking agent to block the remaining non-specific binding sites. **(G, H)** Biosensing was done by adding different concentrations of Ag to the antibody bound to functionalized graphene. **(I)** The sensing capabilities of was monitored by continuously measuring the resistance of graphene channel for different concentrations of Ag using a lock-in amplifier. **(b)** Schematic of binding steps of graphene-Ab conjugation: **(A)** exfoliated graphene before activation. **(B)** EDC-NHS activation of carboxylic groups on graphene. **(C)** Binding on amine group of Ab with the activated carboxylic group of graphene to form graphene-Ab conjugation.

Figure 1a shows the steps involved in the fabrication of GraFET device, activation process, antibody attachment and detection of antigen by monitoring change in electrical characteristics whereas Fig. 1b depicts the steps involved in the binding of graphene-Ab conjugate.

**Figure 2:**
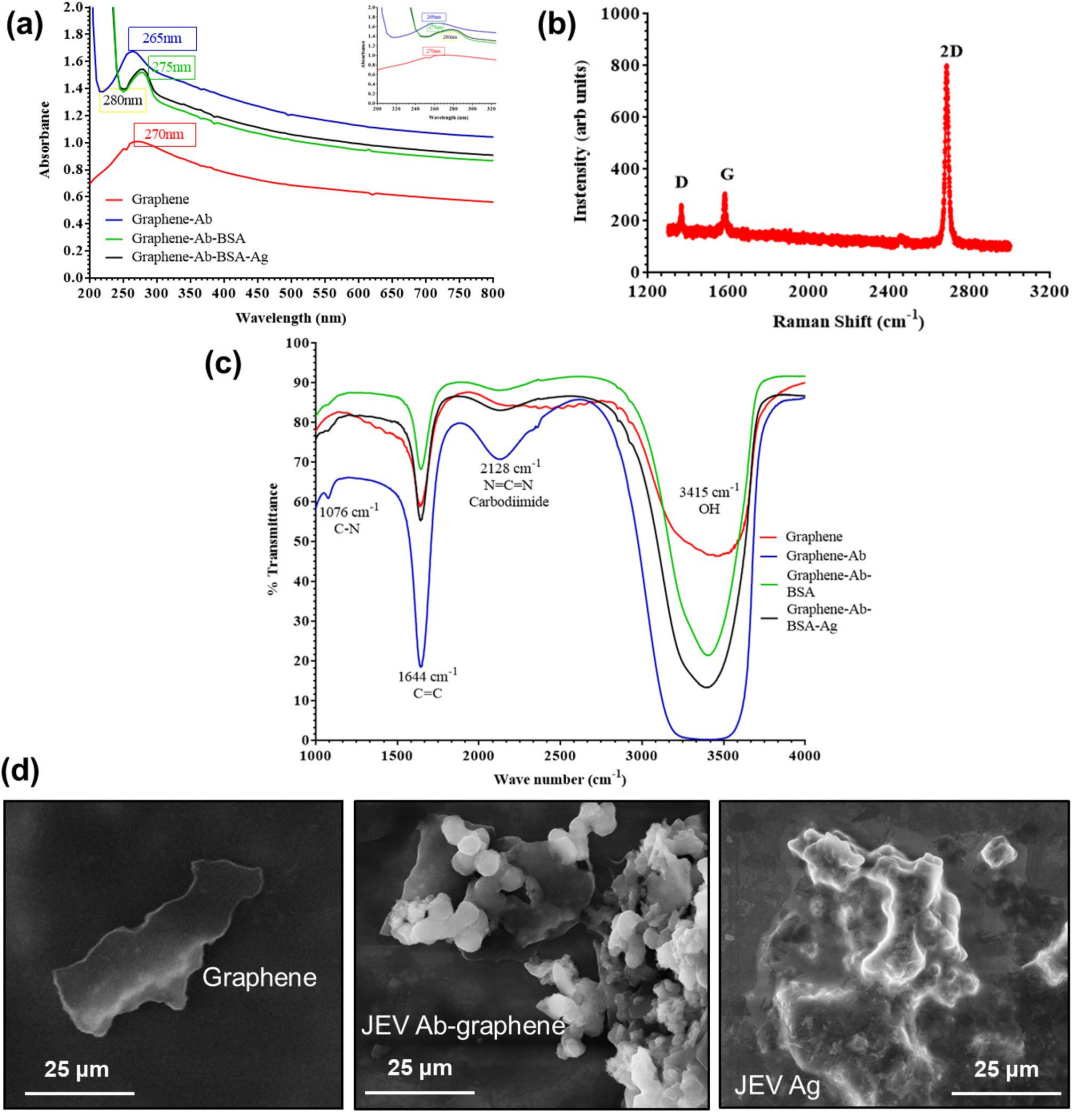
Biophysical characterisation of graphene and its bioconjugate **(a)** UV–Vis spectra showed a peak at 270 nm for graphene, at 265 nm for graphene-Ab, at 275 nm for graphene-Ab-BSA and at 280 nm for graphene-Ab-BSA-Ag. The blue shift can be clearly observed in the inset; **(b)** FT-IR spectrum showed a shared peak at 1644 cm^−1^ (C=C) for graphene as well as graphene-bioconjugates and additional peaks at 1,076 cm^−1^ (C–N) and 2,128 cm^−1^ (N=C=N) for the graphene-Ab (EDC-NHS) conjugate and further steps; **(c)** Raman spectra showed characteristic 2D peak at ~ 2,600 cm^−1^; **(d)** SEM images showed surface morphology of (i) graphene (ii) graphene-Ab (iii) graphene-Ab-Ag.

The binding of graphene with JEV/AIV antibody was ensured by observing various parameters. The UV–Vis spectra (Fig. 2a) which showed a peak for graphene at 270 nm, displayed a blue shift of 5 nm that confirmed the binding of graphene-Ab complex (inset of Fig. 2a). Further addition of Ag led to a red shift of 10 nm (from 265 to 275 nm), as the graphene-Ab bound with specific Ag that confirmed the presence of graphene-Ab-Ag complex. The Raman spectra (Fig. 2b) showed typical single-layer graphene device, with characteristics peaks at 2,600 cm^−1^ and 1,600 cm^−1^. FT-IR spectra in Fig. 2c showed one peak for graphene as well as all graphene-bioconjugates at 1644 cm^−1^ corresponding to C=C bond of graphene molecules which states that structure of graphene does not undergo change during conjugation and further experimentation. On addition of Ab, two additional peaks were observed at 2,128 cm^−1^ (N=C=N) (carbodiimide bond) that confirmed EDC-NHS carbodiimide reaction for activation of carboxyl groups on graphene, and another peak at 1,076 cm^−1^ (C–N) that confirmed binding of amine group of Ab to activated carboxyl group of graphene. Moreover, all three peaks could be observed on addition of BSA and Ag which proved no further changes in the graphene-Ab conjugation. The morphological characteristics were observed by SEM (Fig. 2d) at different stages of conjugation i.e. (i) only graphene, (ii) graphene-Ab, (iii) graphene-Ab-Ag complex, that showed changes in the surface of graphene at each step of immobilization.

### Electrical characterisation of GraFET biosensor

**Figure 3:**
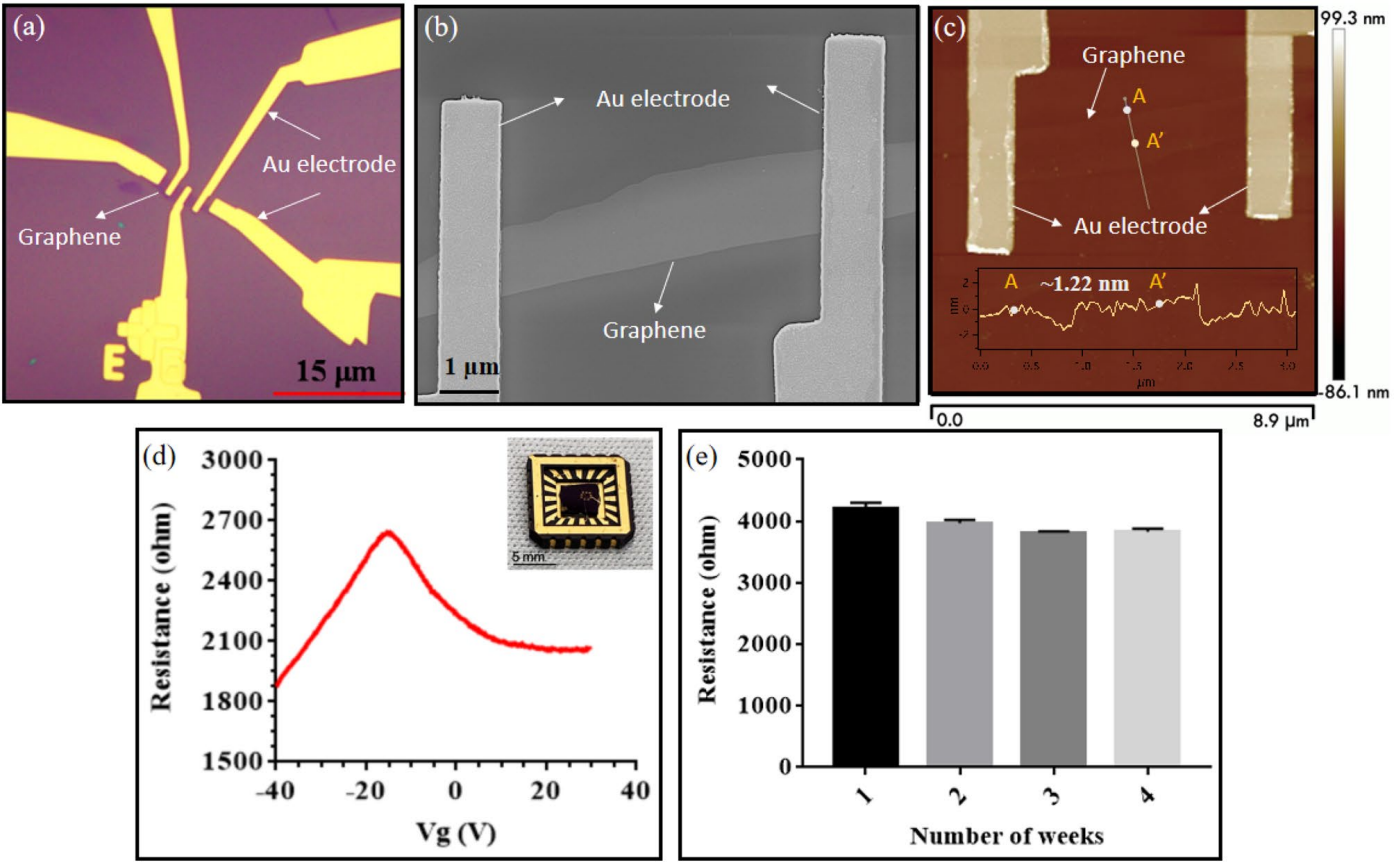
** (a)** Optical micrograph of a typical GraFET device before measurement; **(b)** SEM micrograph of device with the surface of graphene connected with gold electrode; **(c)** AFM image of bare graphene without antibody binding; **(d)** Resistance vs gate voltage plot of a prototypical GraFET device with an inset image of the device; **(e)** Resistance vs time graph monitored over a span of 4 weeks showed negligible change in the current response.

The optical micrograph of a typical GraFET device before measurement was showed in Fig. 3a. The scanning electron and atomic force micrograph of a typical device was depicted in Fig. 3b, c respectively, with the height of the graphene channel to be 1.22 nm. The inset in Fig. 3d showed the image of the packaged device mounted on a ceramic chip-carrier. To check the device characteristics before the measurements, resistance ($R$) vs gate-voltage (${V}_{g}$) measurements were performed, where ${V}_{g}$ was swept at a rate of 600 V/h. The $R$-${V}_{g}$ curve of a prototypical device shown in Fig. 3d, displayed an ambipolar behaviour, where the peak in resistance corresponds to the Dirac point or charge neutrality point. Before the actual measurements were performed, we checked the response of the antibody-antigen combination on a standard Au-FET, which showed negligible change in channel resistance, implying that the Au electrodes used in the FET do not play any role^29^. Additionally, to ensure the stability of our GraFET sensors, we monitored the resistance of the GraFET channel up to four weeks. As can be seen from Fig. 3e, a negligible change in the channel resistance was observed which proved that the FETs were stable against environmental degradation for at least a month.

### Binding, competitive and specificity assay for JEV and AIV

**Figure 4:**
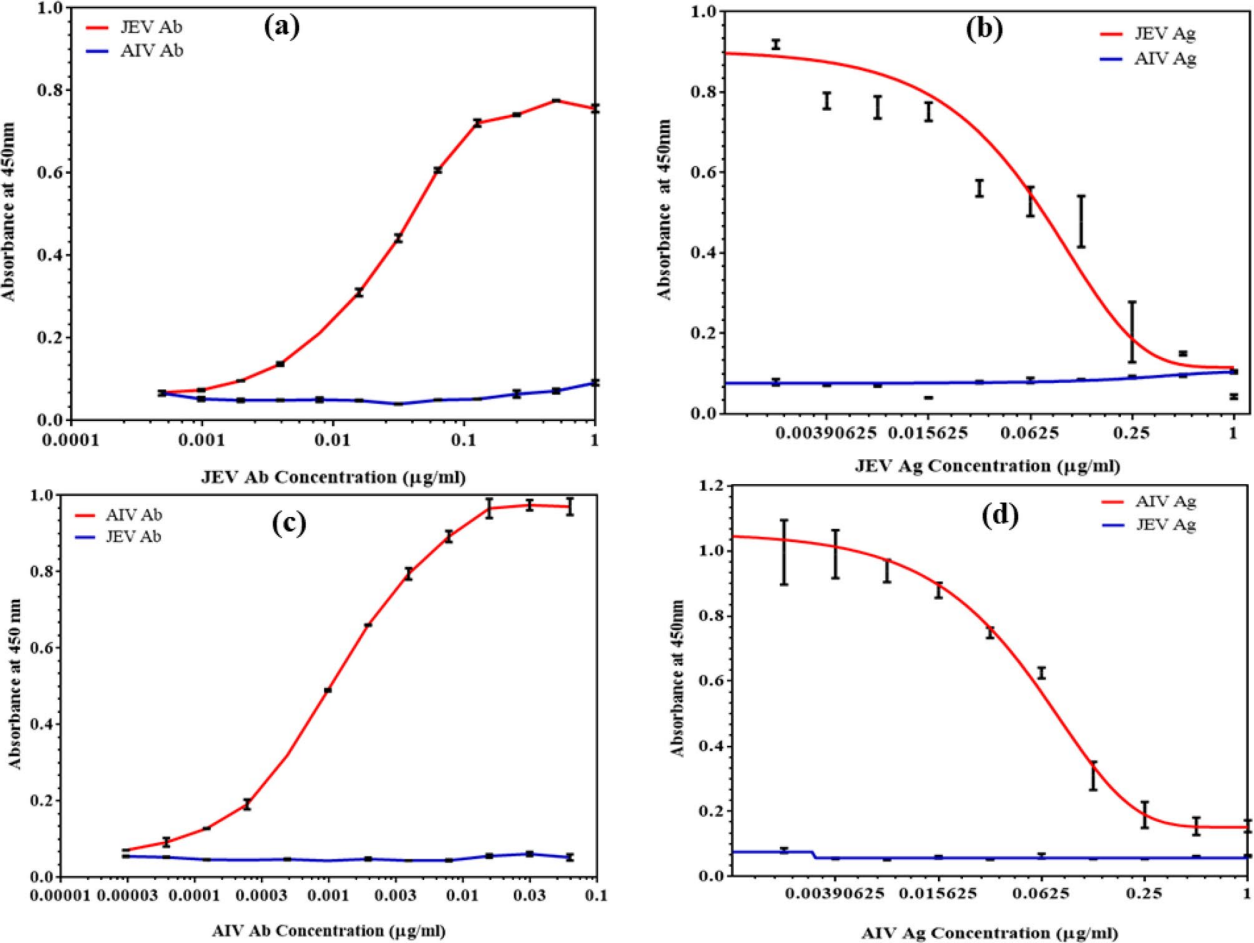
ELISA assays for comparison with the developed biosensor: **(a)** Indirect binding ELISA and specificity assay for JEV, **(b)** Competitive ELISA and specificity assay for JEV, **(c)** Indirect binding ELISA and specificity assay for AIV, **(d)** Competitive ELISA and specificity assay for AIV.

Binding assay for JEV was performed in the range of 1 μg/mL to 0.0019 μg/mL while for AIV from 0.0625 to 0.000031 μg/mL (Fig. 4a, c). The optimum binding was observed from 0.03 to 0.1 μg/mL for JEV, and 0.0003 to 0.01 μg/mL for AIV. Along with the binding assay, specificity assay for binding of JEV Ag with AIV Ab and vice-versa was also carried out (Fig. 4a, c respectively) and it was observed that no cross-reactivity takes place. This shows that using these sets of Ag and Ab will ensure specificity of the developed immunosensor as there are negligible chances of cross-reactivity. For competitive immunoassay, we used 0.1 μg/mL for JEV Ab and 0.01 μg/mL for AIV Ab as fixed concentration, that was used for competitive reaction with a range of antigen from 1 μg/mL to 0.0019 μg/mL. The LOD was found to be 0.25 μg/mL for JEV and 0.031 μg/mL for AIV as shown in Fig. 4b, d. Along with the competitive assay, specificity assay was once again carried out to check for non-specific binding of JEV Ag with AIV Ab and vice-versa (Fig. 4b, d respectively) and it was observed that no cross-reactivity took place. This confirmed that using these sets of Ag and Ab will ensure specificity of the developed immunosensor as there are negligible chances of cross-reactivity.

### Electrochemical performance of developed GraFET sensor for JEV and AIV

**Figure 5:**
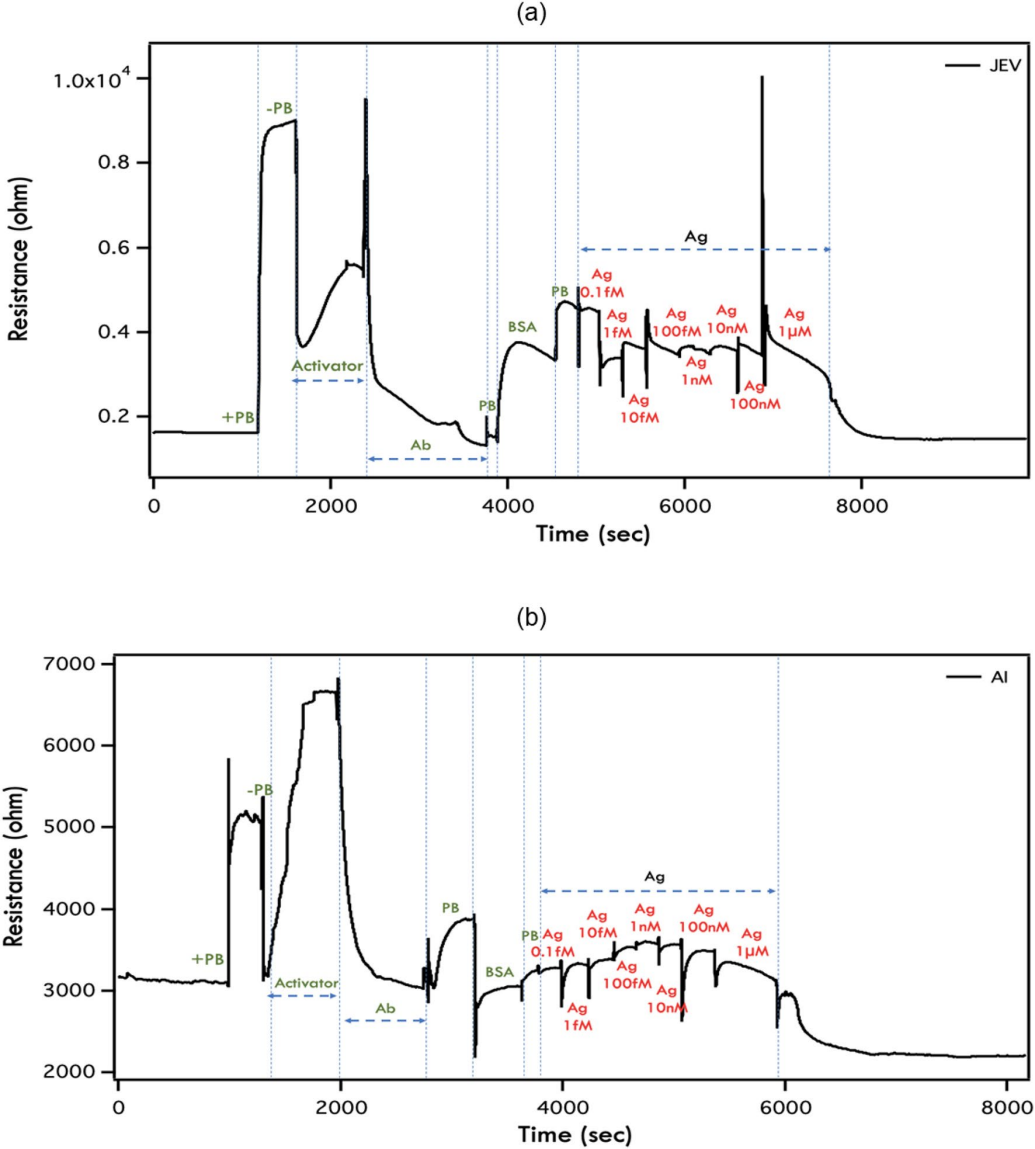
Kinetic response of developed GraFET device of **(a)** Japanese encephalitis virus (JEV) and **(b)** Avian influenza virus (AIV) after addition of specific antigen. The different concentrations of antigen added: (a–h) 1 fM–1µM in 50 mM PB (pH 7.4).

Various GraFET devices were fabricated to monitor the real-time change in resistance with time to examine the device performance for the detection of JEV and AIV, at a constant current of 100 nA and ${V}_{g}=0$ V. The graphene was activated with EDC-NHS which led to the decrease in resistance. The activated graphene channel was then treated with the specific antibody (JEV or AIV) and excess material was washed away. Blocking was performed with BSA to ensure that all the free-sites present in graphene channels were blocked and any further change in resistance that would appear was only due to antibody-antigen interaction. As observed in Fig. 5a,b, there was a clear change in the channel resistance due to a change in the doping profile of graphene with subsequent addition of antigen (JEV and AIV) ranging from 1 fM to 1 µM. As shown in Fig. 5a, after 1 fM addition of antigen, the resistance of graphene channel decreases and at 100 nM of JEV it saturated. However, as shown in Fig. 5b we observed a drop in resistance at 10 nM AIV antigen concentration and saturation at 1 µM. The antibody-antigen binding modifies the local electrostatic environment^48^ which leads to a change in the number density of the graphene channel, manifested as a change in the resistance/conductance during the measurements. The eventual saturation in kinetic response on subsequent addition of antigens beyond a device specific threshold value could be an indicator that all the active sites on the device are occupied. This prevents any further change transfer, and hence any change in the channel resistance/conductance is also absent. The increase or decrease of resistance (or conductance) depends on the type of doping of both the channel and that due to the target-analyte interactions^49^.

For a quantitative analysis of the GraFET response towards sensing after addition of the specific antigen to the antibody bound to graphene, the percentage change in channel resistance was calculated, where resistance in buffer solution (PB) (added just before the antigen addition) was taken as the baseline as shown in Fig. 5a,b. The maximum change in channel resistance calculated for JEV was ~ 10% (Fig. 5a) and for AIV it was ~ 20% (Fig. 5b), for Ag concentrations ranging from 1 fM to 1 µM, which made our GraFET biosensors one of the most sensitive detectors till date in identifying JEV and AIV (as depicted in Table 1). It is more sensitive than the ELISA tests showed in Fig. 4 (a,b for JEV and c,d for AIV). Further improvement in sensitivity is possible by increasing the number of active sites, possibly by using a larger area of graphene. Reproducibility of these devices were carried out in our earlier research work^29^ where different antigen were tested on three separate sensors each and the consistent results proved high reproducibility of this immunosensor. Similarly specificity studies were also carried out in the same research paper^29^ using different antigen. Additionally, these devices are highly stable, and can be integrated into electronic chips for cost effective, on-field detection along with ease of handling.

**Table 1:** Comparison of different types of biosensors developed for the detection of Japanese encephalitis virus and avian influenza virus based on limit of detection (LOD) and range of detection.

Type of biosensor	Limit of detection	Range	References
**Japanese encephalitis virus**			
Silanized interdigitated electrochemical sensor	0.75 μg/mL	1 to 10 μg/mL	^50^
FRET based virus-MIP fluorescent optical sensor	9.6 pM	24 to 960 pM	^51^
MIP silica microspheres based fluorescence optical sensor	0.11 pM	2.4 to 24 pM	^52^
Magnetic MIP based resonance light scattering optical sensor	1.3 pM	–	^53^
Electrochemical sensor using APTES-glutaraldehyde-serum	10 ng/mL	25 ng/mL to 1 μg/mL	^54^
Polyaniline nanowires-based interdigitated platinum electrochemical sensor	< 10 ng/mL	10 to 500 ng/mL	^55^
Gold coated magnetic bead based electrochemical sensor with MWCNT	0.56 ng/mL	0.84 to 11,200 ng/mL	^56^
AuNP based SPCE electrochemical impedimetric electrochemical sensor	167 pfu/mL	500 to 5 × 10^5^ pfu/mL	^57^
AgNP based silanized glass slide optical sensor	12.8 ng/mL	14 to 100 ng/mL	^58^
CNP (from starch NP) based SPCE electrochemical sensor	2 ng/mL	5 to 20 ng/mL	^59^
CNP (from chitosan NP) based SPCE electrochemical sensor	0.36 ng/mL	1 to 20 ng/mL	^60^
GraFET based electrochemical sensor	1 fM	1 fM to 1 μM	Current work
**Avian influenza virus**			
Microgravimetric QCM based piezoelectric sensor	4 virus particles/mL (29.6 ng/mL)	0.02 to 3 HAU	^61^
AuNP based QCM piezoelectric sensor	10^3^ pfu/mL (10 μg/mL)	10^3^ to 10^7^ pfu/mL	^62^
Interdigitated array microelectrode based faradic impedance electrochemical sensor	10^3^ EID_50_/mL	10^3^ to 10^7^ EID_50_/mL	^63^
Portable impedance electrochemical sensor	10^3^ EID_50_/mL	10^2^ to 10^5^ EID_50_/mL	^64^
Nanobeads based QCM piezoelectric sensor	0.128 HAU	0.128 to 12.8 HAU	^65^
Magnetic nanobeads and microfluidic chip with an interdigitated array microelectrode based non-faradic impedance electrochemical sensor	10^2.2^ ELD_50_/mL	10^1.2^ to 10^5.2^ ELD_50_/mL	^66^
EIS based electrochemical sensor	8 ng/mL	0 to 64 ng/mL	^67^
QD-induced FRET based fluorescence sensor	0.5 nM	0.5 nM to 1 μM	^68^
SPR based optical sensor	0.128 HAU	0.128 to 12.8 HAU	^69^
Magnetic nanobeads based QCM piezoelectric sensor	1 HAU	–	^70^
SPR based optical sensor	67 fM	–	^71^
Hydrogel based QCM piezoelectric sensor	0.0128 HAU	0.0128 to 64 HAU	^72^
EIS based universal impedance electrochemical sensor	20 pg/mL	10 to 80 pg/mL	^73^
AgNP based fluorescence optical sensor	10^–13^ g/mL	10^−12^ to 10^−8^ g/mL	^74^
Polypyrrole modified with ferrocene transducer based impedance electrochemical sensor	0.42 nM	5 nM to 1.5 mM	^75^
BaGdF_5_:Yb/Er UCNP-AuNP LRET based optical sensor	7 pM	10 pM to 10 pM	^76^
Co-porphyrins based impedance electrochemical genosensor	21 fM	10 to 80 fM	^77^
Isothermal exponential amplification coupled with hybridization chain reaction of DNAzyme nanowires based electrochemical impedance genosensor	9.4 fM	50 to 100 pM	^78^
AuCNT and QD based PAFI optical sensor	0.1 pg/mL 50 PFU/mL	50 to 10,000 PFU/mL	^79^
Bifunctional magnetic beads based electrochemical sensor	6.8 pg/mL	0.01 to 20 ng/mL	^80^
Ag@SiO_2_ NP based MEF optical sensor	2 ng/mL (buffer) 3.5 ng/mL (serum)	2 to 100 ng/mL	^81^
AuNP on carbon chips based amperometric electrochemical genosensor	100 fM	100 fM to 100 pM	^82^
AgNP coated graphene based impedance electrochemical sensor	1.6 pg/mL	1.6 × 10^–3^ to 16 ng/mL	^83^
Phase-intensity SPR based optical sensor	193.3 ng/mL	–	^84^
GraFET based electrochemical sensor	10 fM	1 fM to 1 μM	Current work

## Materials and methods

### Materials and reagents

Single crystals of Kish graphite were obtained from Covalent Materials Corp. (Tokyo, Japan) for exfoliation while 99.9% pure gold wire and chromium pellets were obtained from Kurt J. Lesker Co. (Clairton, PA, USA) for thermal evaporation. Graphene was exfoliated using Scotch tape (3 M). 285 nm SiO_2_/Si substrate was obtained from Nova Electronic Materials (TX, USA). The JEV Ag and anti-mouse monoclonal anti-JEV Ab was purchased from the Native Antigen Company (Oxford, UK) whereas the anti-rabbit monoclonal anti-AIV Ab and AIV Ag were purchased from Merck (Mumbai, India). Goat anti-mouse Horseradish Peroxidase (HRP) conjugated secondary Ab and Goat anti-rabbit Horseradish Peroxidase (HRP) conjugated secondary Ab was procured from Proteintech (Rosemont, Illinois, USA). 1-ethyl-3-(3-dimethylaminopropyl)carbodiimide hydrochloride (EDC), N-hydroxysulfosuccinimide (sulfo-NHS) and Bovine Serum Albumin (BSA) were purchased from Sigma-Aldrich (Delhi, India). Poly(methyl methacrylate) (PMMA) 495 and 950 were bought from Micro Chem Corp. (Newton, MA, USA). Tetramethylbenzidine (TMB) Substrate was obtained from HiMedia (Mumbai, India). All the reagents used were of high quality analytical grade and all solutions and buffers were prepared using double distilled water.

### Apparatus

UV–Vis and FT-IR spectra were acquired on Systonic S-924 Single-Beam UV–Vis Spectrophotometer (Delhi, India) and Thermo Scientific-Nicolet iS50 FT-IR (Bangalore, India) respectively. The Raman spectrum was obtained using LabRAM HR Horiba Jobin–Yvon Raman Spectroscope (Bangalore, India). Surface morphology was examined using Scanning Electron Microscope Hitachi S-3400N and Zeiss Gemini Ultra 55 (Bangalore, India). Optical was performed in Microscope Olympus BX51 (Bangalore, India), and AFM was in Bruker Dimension Icon (Bangalore, India). ELISA readings were scanned in Perkin Elmer Lambda 25 Multi-scan Spectrophotometer (Bangalore, India) and Nunc 96-multiwell ELISA plates were used. All electrical measurements were performed using the SRS 830 lock-in amplifier at a carrier frequency of 227.7 Hz. The gate voltage for obtaining the transfer characteristics was applied using Keithley 2,400 Source Meter. All experiments were performed at room temperature (25 °C) unless stated otherwise.

### Bioconjugation of graphene-antibody and biophysical characterization

To immobilize antibodies on graphene, the graphene must first be activated using carbodiimide chemistry. 0.5 mg of graphene was added to 75 μm EDC and 75 μm sulfo-NHS (total volume 1 mL) and gently stirred for 2 h at RT. Here EDC in conjugation with NHS allows a two-step coupling reaction by activating the carboxyl groups for conjugation. The activated graphene complex was then centrifuged for 15 min at 10,000 rpm at 4 °C following which the pellet was resuspended in 1 mL phosphate buffer (PB) (50 mM, pH 7.4). Anti-JEV and anti-AIV monoclonal antibodies were drop-wise added to activated graphene complex, separately, for 30 min at RT and then left overnight for incubation at 4 °C. The unbound sites of the graphene-Ab complex were blocked using 1% BSA in 50 mM PB (pH 7.4) for 2 h at RT. Antigen (JEV and AIV separately) was added to the graphene-Ab complex after blocking, and incubated for 2 h at RT. To confirm the above binding steps, characterisation of each step was carried out using Single-beam UV–Vis spectrometer in the range of 190–800 nm, Fourier Transformed Infra-Red (500–4,000 cm^−1^), Raman Spectroscopy (1,300–3,000 cm^−1^) and Scanning Electron Microscope (morphological analysis). Quorum SC7620 sputter coater was used to electro-activate the samples for SEM imaging by applying a coat of gold.

### Binding, competitive and specificity ELISA for JEV and AIV

Using the antigen and antibodies procured for JEV and AIV, both binding and competitive ELISA were carried out to check the sensitivity of immunoassay and later compared to the sensitivity of the developed GraFET sensor. For standardisation of the antibody concentration to be used in competitive assay, indirect binding ELISA was carried out. 96-well NUNC ELISA plates were coated with 100 μL of JEV and AIV antigen separately [0.25 μg/mL in carbonate buffer (pH 9.6)] (optimal standardised concentration) and incubated O/N at 4 °C. The plates were washed thrice with 0.02% Phosphate Buffer Saline-Tween20 (PBS-T) followed by blocking using 2% Phosphate Buffer Saline-Skimmed Milk (PBS-M) (250 μL/well) in at 37 °C for 1 h. Plates were washed thrice with 0.02% PBS-T. twofold dilutions in 0.1% PBS-M of anti-JEV monoclonal Ab (100 μL/well) were added in triplicates ranging from 1 to 0.0019 μg/mL to the JEV Ag coated plate. Similarly, twofold dilutions in 0.1% PBS-M of anti-AIV monoclonal Ab (100 μL/well) were added in triplicates ranging from 0.0625 to 0.000031 μg/mL to the AIV Ag coated plate. The plates were incubated at 37 °C for 2 h followed by washing thrice with 0.02% PBS-T. Goat anti-mouse HRP-conjugated 2° Ab and Goat anti-rabbit HRP-conjugated 2° Ab (1:2,500 dilution in 0.1% PBS-M) were added to JEV and AIV plates respectively (100 μL/well) and incubated for 1 h at 37 °C. The plates were again washed thrice with 0.02% PBS-T. 100 μL TMB substrate (1×) was added to each well and allowed to incubate for 15 min at RT. 100 μL stop solution (1 N H_2_SO_4_) was added to stop the reaction. Readings were taken at 450 nm in Perkin Elmer Lambda 25 Multi-scan Spectrophotometer. After carrying out the binding assay, the same experiment was carried out as above using AIV Ab on JEV Ag coated plate and JEV Ab on AIV Ag coated plate. All the concentrations and experimental steps were followed the same as the above binding assay. This cross reactivity test was carried out to check the specificity of the Ag-Ab interaction and reading were taken at 450 nm in Perkin Elmer Lambda 25 Multi-scan Spectrophotometer.

From the above binding assay, the parameters and concentrations were standardised for competitive ELISA for both JEV and AIV. The initial steps remained the same as the above indirect ELISA. After blocking and washing, different Ag-1°Ab (JEV/AIV) were added to the plates (100 μL/well) where the 1°Ab concentration remained constant (0.1 μg/mL for JEV and 0.01 μg/mL for AIV as standardised in the indirect ELISA) and the Ag concentrations were diluted twofold in 0.1% PBS-M ranging from 1 μg/mL to 0.0019 μg/mL. After 2 h incubation at 37 °C the remaining steps i.e. 2°Ab, washing, TMB and stop solution remain the same as above. Readings were taken at 450 nm in Perkin Elmer Lambda 25 Multi-scan Spectrophotometer. After carrying out the competitive assay, the same experiment was carried out as above using AIV Ab-JEV Ag conjugate on JEV Ag coated plate and JEV Ab-AIV Ag conjugate on AIV Ag coated plate. All the concentrations and experimental steps were followed the same as the above competitive assay. This cross reactivity test was carried out to check the specificity of the Ag-Ab interaction and reading were taken at 450 nm in Perkin Elmer Lambda 25 Multi-scan Spectrophotometer.

### Fabrication of GraFET sensor

Graphene was mechanically exfoliated from bulk graphite using the conventional scotch-tape method^85^ with 3 M magic tape, on RCA-cleaned hot SiO_2_/Si substrate, where the 285 nm SiO_2_ on Si functions as the back-gate dielectric. A high-resolution (1000×) optical microscope (Olympus BX51) was used to locate and select the desired graphene sheets. The substrates were spin-coated with PMMA 495 and PMMA 950 positive-resist, and baked at 150 °C. Electron beam lithography (Raith Pioneer) was performed to define the electrode patterns, which were developed using 1:3 mixture of methyl isobutyl ketone (MIBK) and isopropyl alcohol (IPA). After development, 5 nm thick Cr, and 50 nm thick Au layers were thermally evaporated to form the source-drain contacts followed by the lift-off process. Substrates were fixed onto a ceramic chip-carrier using silver-epoxy paste, and wire-bonded (TPT HB05 wire bonder) to the active contact pads.

### Electrochemical performance of GraFET sensor

The JEV and AIV antibodies were pre-activated separately by EDC/NHS and immobilised on the surface of FET by being allowed to bind to the graphene for 30 min in 50 mM Phosphate Buffer (PB) at pH 7.4. Excess unbound Abs were washed off using 50 mM PB (pH 7.4). 1% BSA in 50 mM PB (pH 7.4), was used to block any non-specific sites. Different concentrations (1 fM to 1 μM) of JEV/AIV Ag were prepared in 50 mM PB (pH 7.4) to check the sensitivity of the fabricated FET biosensor. Measurements were performed and the change in the resistance was recorded at each stage by passing a constant current circuit of 100 nA through the FET device.

## Conclusions

From our study, it can be concluded that GraFETs are an excellent tool for early detection of JEV and AIV. The developed biosensor proved to be highly sensitive with a detection range of 1 fM to 1 μM and limit of detection of 1 fM for JEV and 10 fM for AIV. This proved that graphene is an excellent choice for real-time sensing of low concentration of JEV/AIV antigen in the initial stages of the infection itself resulting in rapid diagnosis. The major advantage of this device over conventional JEV/AIV diagnosis methods, besides sensitivity and specificity, is quick response time and high POC potential. However, the miniaturization of the device for on-site applicability in the field would be expensive especially since each device can be used only once and can not be repeated. Alternate cost-effective fabrication processes need to be developed so that this device can be used as a Point of Care diagnostic platform for JEV/AIV as well as other diseases.
